# A machine learning based radiomics approach for predicting No. 14v station lymph node metastasis in gastric cancer

**DOI:** 10.3389/fmed.2024.1464632

**Published:** 2024-10-18

**Authors:** Tingting Ma, Mengran Zhao, Xiangli Li, Xiangchao Song, Lingwei Wang, Zhaoxiang Ye

**Affiliations:** ^1^Department of Radiology, Tianjin Cancer Hospital Airport Hospital, Tianjin, China; ^2^Department of Radiology, Tianjin Medical University Cancer Institute and Hospital, Tianjin, China; ^3^National Clinical Research Center for Cancer, Tianjin, China; ^4^Tianjin’s Clinical Research Center for Cancer, Tianjin, China; ^5^The Key Laboratory of Cancer Prevention and Therapy, Tianjin, China; ^6^Health Management Center, Weifang People’s Hospital, Weifang, China

**Keywords:** radiomics, computed tomography, gastric cancer, lymph node metastasis, 14v station

## Abstract

**Purpose:**

To evaluate the potential of radiomics approach for predicting No. 14v station lymph node metastasis (14vM) in gastric cancer (GC).

**Methods:**

The contrast enhanced CT (CECT) images with corresponding clinical information of 288 GC patients were retrospectively collected. Patients were separated into training set (*n* = 202) and testing set (*n* = 86). A total of 1,316 radiomics feature were extracted from portal venous phase images of CECT. Seven machine learning (ML) algorithms including naïve Bayes (NB), *k*-nearest neighbor (KNN), decision tree (DT), logistic regression (LR), random forest (RF), eXtreme gradient boosting (XGBoost) and support vector machine (SVM) were trained for development of optimal radiomics signature. A combined model was established by combining radiomics with important clinicopathological factors. The diagnostic ability of the signature and model were evaluated.

**Results:**

LR algorithm was chosen for signature construction. The radiomics signature exhibited good discrimination accuracy of 14vM with AUCs of 0.83 in the training and 0.77 in the testing set. The risk of 14vM showed significant association with higher radiomics score. A combined model exhibited increased predictive ability and good agreement in the training (AUC = 0.87) and testing (AUC = 0.85) sets.

**Conclusion:**

The ML-based radiomics model provided a promising image biomarker for preoperative detection of 14vM and may help the surgeon to decide whether to add 14v dissection to lymphadenectomy.

## Introduction

Lymph node metastasis (LNM) significantly contributes to the poor prognosis of patients with gastric cancer (GC) (1). Adequate lymph node dissection is curial for the successful management of GC. According to the recommendation of NCCN and GC treatment guidelines, D2 lymphadenectomy is now the standard procedure of GC surgery (2, 3). Superior mesenteric lymph nodes, referred to as No. 14v, are involved in the lymphatic drainage of the lower stomach (4). Nevertheless, the necessity of extended D2 lymphadenectomy with No. 14v dissection is still a hot point for GC surgery. Whether GC patients can benefit from No. 14v dissection remains controversial. It is well established that patients with No. 14v metastasis (14vM) are related to poor prognosis, similar to those of the M1 stage (5, 6). Upon the presence of undetectable microscopic metastases in the No. 14v station, systemic dissection of this area may prevent the metastatic process to adjacent retroperitoneal lymph nodes and reduce recurrence risk (7). Although the rate of 14vM is extremely low in early-stage GC at 0–1.3%, it rises up to 19.7% in advanced GC (6, 8). Several studies have reported that extended D2 lymphadenectomy with 14v dissection was related to better survival in advanced distal GC patients (7–9). However, No. 14v dissection may increase the risk of neighboring vessel injuries. Therefore, the third edition of Japanese gastric cancer treatment guidelines removed No. 14v lymphadenectomy from D2 lymphadenectomy (3). Thus, it is of great help to guide surgeons on whether to involve No. 14v dissection in D2 lymphadenectomy if 14vM can be accurately determined preoperatively.

At present, CT is the routine technique for preoperative LNM diagnosis in GC. However, conventional CT relies only on morphological features and enhancement patterns to determine lymph node status, which leads to low sensitivity (10, 11). Endoscopic ultrasonography (EUS) determines LNM by evaluating the change of size, morphology, and internal echogenicity of the lymph nodes. However, EUS is susceptible to interference by air-containing tissues, and difficult to identify microscopic metastases that do not cause morphologic changes in lymph nodes. Although EUS-guided fine needle aspiration is able to improve LNM diagnostic accuracy, the invasiveness limits its application. Moreover, diagnostic performance of EUS depends greatly on the experience of the operator (12, 13). PET/CT can provide both morphological and metabolic information, allowing it to be an important preoperative staging tool for GC (14). However, two key factors lead to the low sensitivity of PET/CT for detecting LNM. Metastatic lymph nodes with diameter less than 1 cm or with low expression of Glut-1 are hard to detect by PET/CT. In addition, lymph node micrometastasis was reported to be occurred in 10%–41.7% of GC patients, and it is impossible for conventional diagnostic imaging tools to detect micrometastasis (15).

Radiomics, a groundbreaking method, is significantly influencing and transforming the field of medical imaging in clinical settings. Radiomics offer novel perspectives for processing image data and can convert images into numerical data, enabling the detection of details and variations in tumors that are imperceptible to the naked eye when using traditional CT scans (16). GC patients can benefit from radiomics in multiple facets, including diagnosis, predicting metastatic risk, survival, as well as treatment response (17–19). Moreover, radiomics can be used to predict LNM in several tumors, including GC (20–23). However, most of the radiomics models proposed by previous studies can only be used for identifying the presence of LNM in patients but not for detecting LNM in a specific region. Herein, our aim is to investigate the radiomics approach potential for predicting 14vM in GC using machine learning (ML) algorithms. A comprehensive model incorporating radiomics and clinicopathological variables was developed and evaluated.

## Materials and methods

### Study population

**Figure 1:**
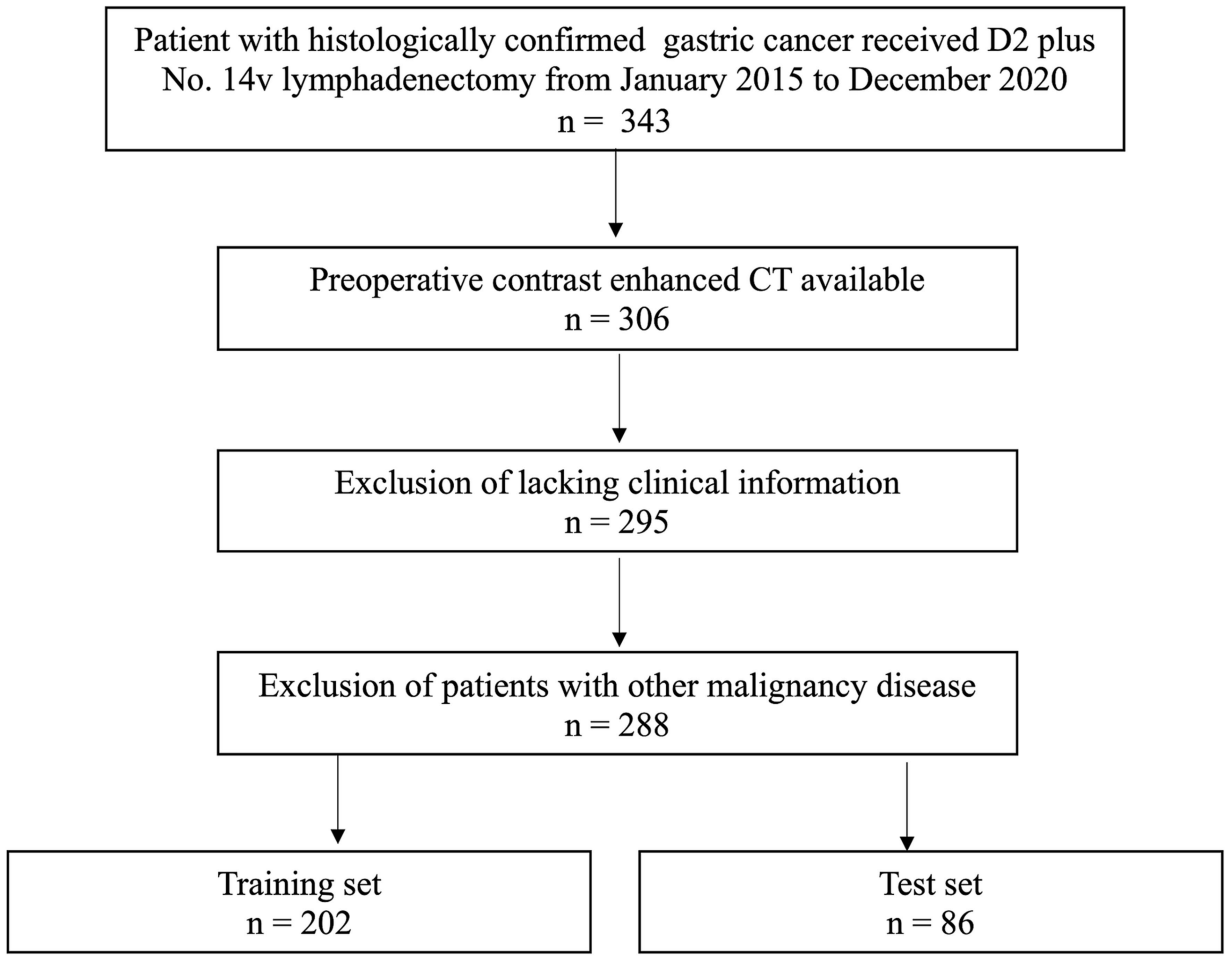
Recruitment pathways for patients.

Between January 2015 to December 2020, 288 consecutive GC patients were included. The inclusion criteria: (1) patients received No. 14v lymphadenectomy adding to standard D2 lymphadenectomy; (2) received contrast enhanced CT (CECT) before surgery; (3) imaging quality meeting the analysis requirements. The exclusion criteria: (1) incomplete clinical records; (2) patients who had pre-CT treatment; (3) patients having a malignancy history. The patients were separated into a training (*n* = 202) and a testing (*n* = 86) sets in a 7:3 ratio based on the diagnosis time. According to the AJCC Staging Manual, 8th Edition, pathologic stages were assigned. The study was approved by our institutional ethical review board, and informed consent form was waived (Ek2020125). Figure 1 shows the patient recruitment process.

### CT image acquisition protocol

Discovery CT750 HD (GE Medical Systems, Milwaukee, Wisconsin) or Somatom Sensation 64 scanner (Siemens Medical Solutions, Forchheim, Germany) was utilized for performing contrast-enhanced abdominal CT. Before undergoing a CT scan, the patient was given 500–1,000 mL of water orally for the purpose of distending the stomach. The parameters were as follows: 120 kVp tube voltage, 150–200 mA tube current; field of view, 350 mm × 350 mm; matrix, 512 × 512; images reconstruction section thickness: 1.25 or 1.5 mm. Contrast material (2.5 mL/s, 1.2 mL/kg; Omnipaque 300, GE Healthcare, Chicago, Illinois) was injected intravenously using a syringe pump, and arterial phase images were acquired 20 s later; after a 60 s delay, the portal venous phase was acquired, exporting the images in the Digital Imaging and Communications in Medicine (DICOM) format.

### Lesion segmentation, feature extraction and signature building

The volume of interest (VOI) was delineated for each lesion using 3D Slicer software (5.0.2). One reader (7 year-interpretation experience in abdominal CT imaging, TM) independently performed segmentation of all tumors. PyRadiomics 2.2.0 was applied for feature extraction (24). From original and filtered images, 1,316 features were exacted and classifying as first-order statistics, shape, gray level dependence matrix (GLDM), gray level size zone matrix (GLSZM), neighbouring gray tone difference matrix (NGTDM), gray level run length matrix (GLRLM) and gray level co-occurrence matrix (GLCM).

To guarantee the stability of the chosen features, interclass correlation coefficients (ICCs) were calculated to analysis the intraobserver and interobserver accessions. Thirty patients were randomly selected from the training set, and VOIs were draw independently by two readers. The features with ICCs >0.85 were considered stable and kept. Then, the Mann–Whitney *U* test identified features with significant differences between the two groups. Finally, the least absolute shrinkage and selection operator (LASSO) model was utilized to identify best features for signature building (25). After Lasso feature screening, seven ML algorithms including *k*-nearest neighbor (KNN), random forest (RF), support vector machine (SVM), decision tree (DT), eXtreme gradient boosting (XGBoost), naïve Bayes (NB) and logistic regression (LR) were utilized to construct radiomics signature. We adopt 5-fold cross verification to obtain the final signature.

### Model establishment and evaluation

**Figure 2:**
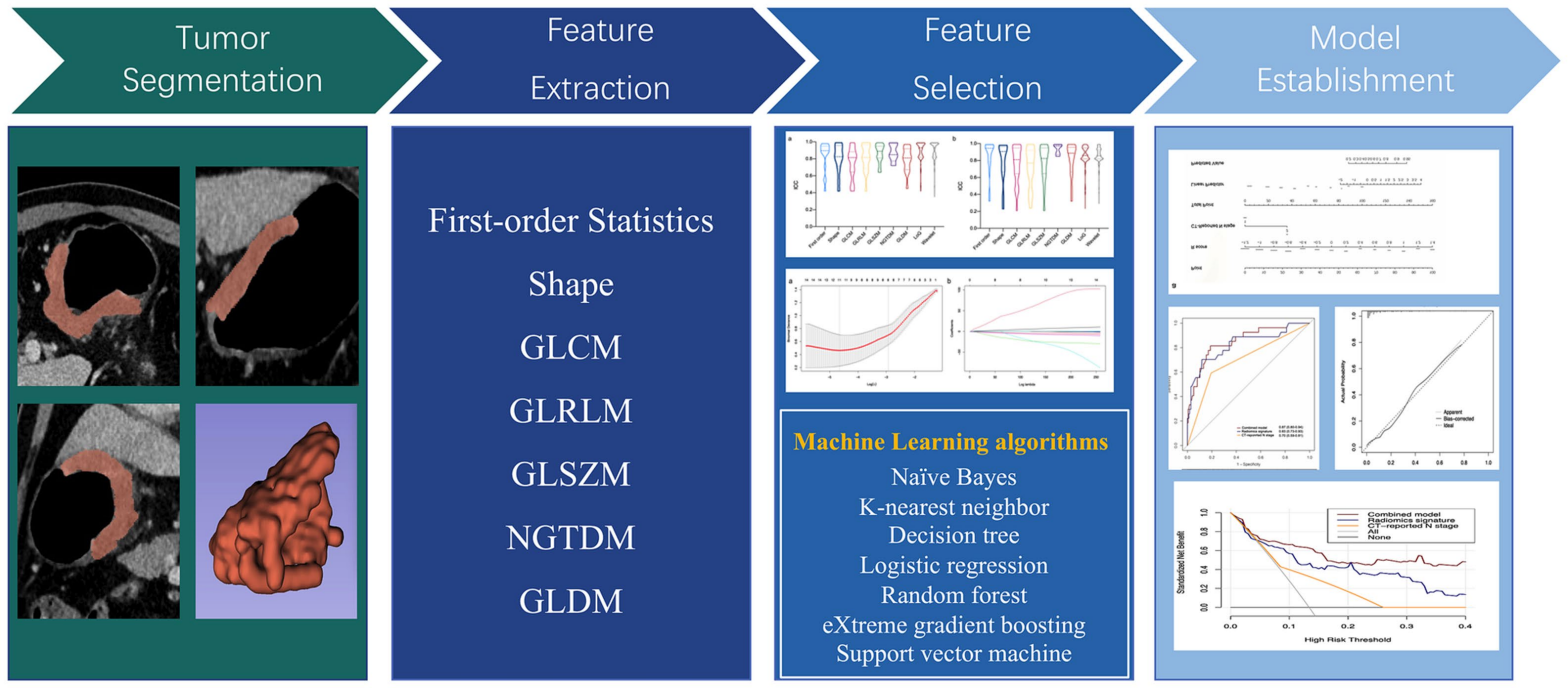
Flowchart of study design.

Multivariable logistic regression analysis was performed to select independent predictors of 14vM, based on which a combined model was constructed. The AUC was utilized to evaluate and compare the diagnostic ability of radiomics signature, clinical predictor and the combined model. Moreover, a nomogram was developed to facilitate the clinical application. The performance of the model was validated in testing set. Figure 2 shows the flowchart of the overall radiomics procedure.

### Statistical analysis

Segmentation agreement of interobserver was analyzed by Dice similarity coefficient. The fitness of the model was evaluated by drawing calibration curves and Hosmer–Lemeshow analysis. Decision curve analysis was mapped out to assess the clinical utility of predictive model in the entire cohort. The *t*-test or the Mann–Whitney test was utilized for continuous variables. The chi-squared test was utilized for categorical variables. All statistical analyses were performed with R (version 3.4.2).

## Results

### Clinical information

The study enrolled 288 patients including 178 males and 110 females (median age, 62 and 56 years; interquartile range, 55–66 and 47–63 years, respectively). The training set contained 27 (13.4%) 14vM+ patients, while the testing set contained 12 (14.0%) 14vM+ patients. In both sets, the 14vM rate was positively associated with a higher LNM category (both CT-reported and pathological N stage). In the training set, the 14vM rate was positively related to the tumor invasion depth (pathological T stage, *p* = 0.03). However, in the testing set, although the proportion of 14vM+ in pT_3–4_ stage was higher than that in pT_1–2_ stage, no significant difference was observed (*p* = 0.06). The 14vM+ and 14vM− groups exhibited no significant difference in age, gender, differentiation status, and four serum biomarker levels. Detailed information of patients was shown in Table 1.

**Table 1:** Characteristics of the study population.

Variable	Training set (*n* = 202)			Testing set (*n* = 86)		
	14vM− (*n* = 175)	14vM+ (*n* = 27)	*p*	14vM− (*n* = 74)	14vM+ (*n* = 12)	*p*
Age			0.66			0.30
<65	136 (77.7)	22 (81.5)		54 (73.0)	7 (58.3)	
≥65	39 (22.3)	5 (18.5)		20 (27.0)	5 (41.7)	
Gender			0.51			0.13
Male	115 (65.7)	16 (44.4)		38 (51.4)	9 (75.0)	
Female	60 (34.3)	11 (55.6)		36 (48.6)	3 (25.0)	
Tumor site			0.04			0.03
Upper-middle	42 (24.0)	6 (22.2)		6 (8.1)	2 (16.7)	
Lower	62 (35.4)	16 (59.3)		31 (41.9)	9 (75.0)	
Overlap	71 (40.6)	5 (18.5)		37 (50.0)	1 (8.3)	
Pathologic T stage			0.03			0.06
T_1–2_	48 (27.4)	2 (7.4)		26 (35.1)	1 (8.3)	
T_3–4_	127 (72.6)	25 (92.6)		48 (64.9)	11 (91.7)	
Pathologic N stage			<0.01			0.02
N_0–1_	102 (58.3)	6 (11.1)		39 (52.7)	2 (16.7)	
N_2–3_	73 (41.7)	24 (88.9)		35 (47.3)	10 (83.3)	
CT reported N stage			<0.01			<0.01
N_0–1_	141 (80.6)	1 (40.7)		59 (79.7)	5 (41.7)	
N_2–3_	34 (19.4)	16 (59.3)		15 (20.3)	7 (58.3)	
Differentiation			0.09			0.54
Well-moderate	32 (18.3)	1 (3.7)		13 (17.6)	3 (25.0)	
Poor	143 (81.7)	26 (96.3)		61 (82.4)	9 (75.0)	
CEA			0.61			0.80
≥5.0 μg/mL	20 (11.4)	4 (14.8)		8 (10.8)	1 (8.3)	
<5.0 μg/mL	155 (88.6)	23 (85.2)		66 (89.2)	11 (91.7)	
CA19-9			0.22			0.43
≥27 U/mL	34 (19.4)	8 (29.6)		12 (16.2)	3 (25.0)	
<27 U/mL	141 (80.6)	19 (70.4)		62 (83.8)	9 (75.0)	
CA242			0.51			0.90
≥20 U/mL	24 (13.7)	5 (18.5)		7 (9.5)	1 (8.3)	
<20 U/mL	151 (86.3)	22 (81.5)		67 (90.5)	11 (91.7)	
CA72-4			0.14			0.68
≥6.9 U/mL	47 (26.9)	11 (40.7)		14 (18.9)	1 (8.3)	
<6.9 U/mL	128 (73.1)	16 (59.3)		60 (81.1)	11 (91.7)	

14vM, No. 14v station lymph node metastasis; CEA, carcinoembryonic antigen; CA, carbohydrate antigen.

### Feature selection and signature development

**Figure 3:**
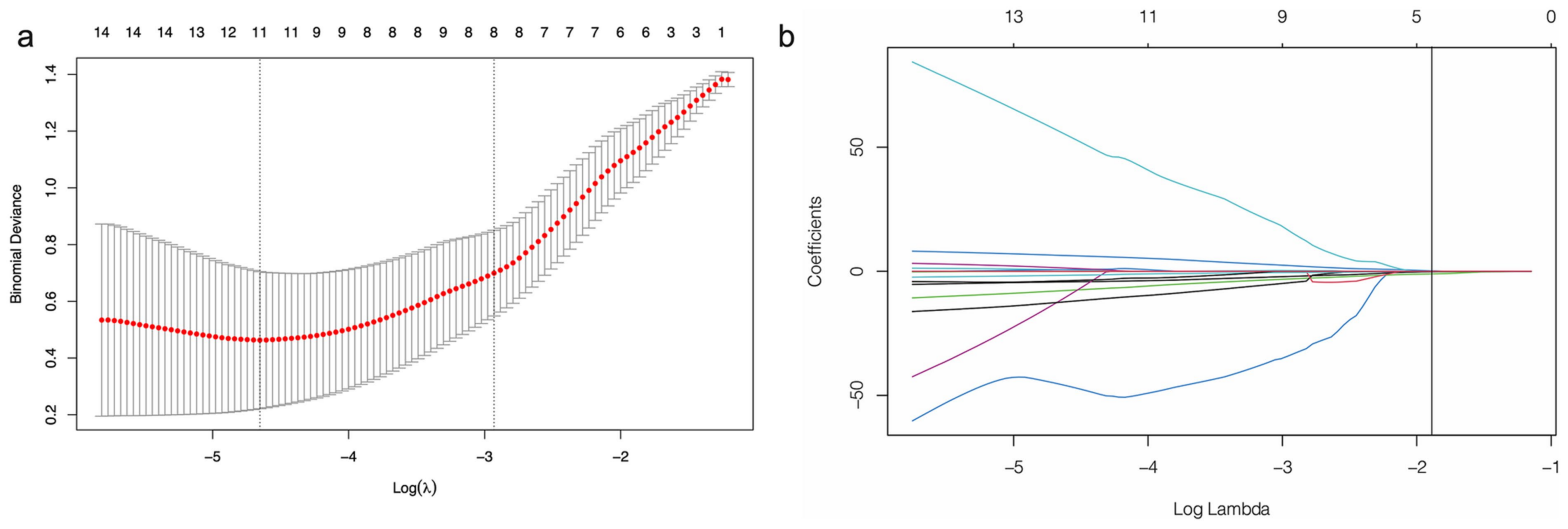
Feature selection using least absolute shrinkage and selection operator (LASSO) logistic regression. (a) Selection of tuning parameter (*λ*) in the LASSO model via 10-fold cross-testing based on minimum criteria. The AUC curve was plotted against log (*λ*). Dotted vertical lines were drawn at the optimal values by using the minimum criteria and the 1 standard error of the minimum criteria (the 1-standard error criteria). (b) LASSO coefficient profiles of the selected features. A vertical line was plotted at the optimal *λ* value, which resulted in seven features with nonzero coefficients.

The Dice similarity coefficient was 0.85, indicating that the readers had good consistence of segmentation. The details of ICCs analysis were shown in Supplementary Figure S1. Out of the 1,316 features retrieved from the training set images, 955 were excluded due to ICCs below 0.85. Among the remaining 361features, 96 features showed significant differences between the 14vM− and 14vM+ groups. These 96 features were putted into the LASSO algorithm. The radiomics signature was constructed based on 7 features with non-zero coefficients (Figure 3). Subsequently, seven other ML models were also trained to determine the best classifier algorithms The AUCs of SVM, DT, KNN, NB, LR, RF and XGBoost models were 0.79, 0.76, 0.80, 0.81, 0.78, 0.83, and 0.69, respectively. The detailed performance of ML models was shown in Table 2. Therefore, LR model was used for developing the signature. The *R*-score calculation formula is as follows:


$$\begin{array}{c} R\mathrm{-score}\;=\;-5.138\;+\;0.357\;\times \;\mathrm{Maximum}\;3D\;\mathrm{diameter}\;+\;1.028\\ \;\times \;\mathrm{log-sigma-1-0-mm-3D-NGTDM-Busyness}\;+\;0.0324\\ \;\times \;\mathrm{wavelet-HHL-NGTDM-Coarseness}\;-\;0.2512\\ \;\times \;\mathrm{GLRLM-Gray}\;\mathrm{Level}\;\mathrm{NonUniformity}\;\mathrm{Normalized}\\ \;+\;0.0267\;\times \;\mathrm{wavelet-LLH-firstorder-Skewness}\;+\;0.621\\ \;\times \;\mathrm{wavelet-LLH-firstorder-Kurtosis}\;-\;1.416\\ \;\times \;\mathrm{wavelet-LLH-GLRLM-RunEntropy} \end{array}$$

**Table 2:** Predictive performances of different machine learning classifiers.

Model	AUC	Accuracy	Sensitivity	Specificity
SVM	0.79	0.75	0.78	0.73
RF	0.76	0.68	0.72	0.67
DT	0.80	0.70	0.69	0.71
KNN	0.81	0.73	0.66	0.78
NB	0.78	0.71	0.65	0.76
LR	0.83	0.86	0.70	0.88
XGBoost	0.69	0.65	0.71	0.60

SVM, support vector machine; RF, random forest; DT, decision tress; KNN, *k*-nearest neighbor; NB, naïve Bayes; LR, logistic regression; XGBoost, eXtreme gradient boosting; AUC, area under the receiver operating characteristic.

### Performance of radiomics signature

**Figure 4:**
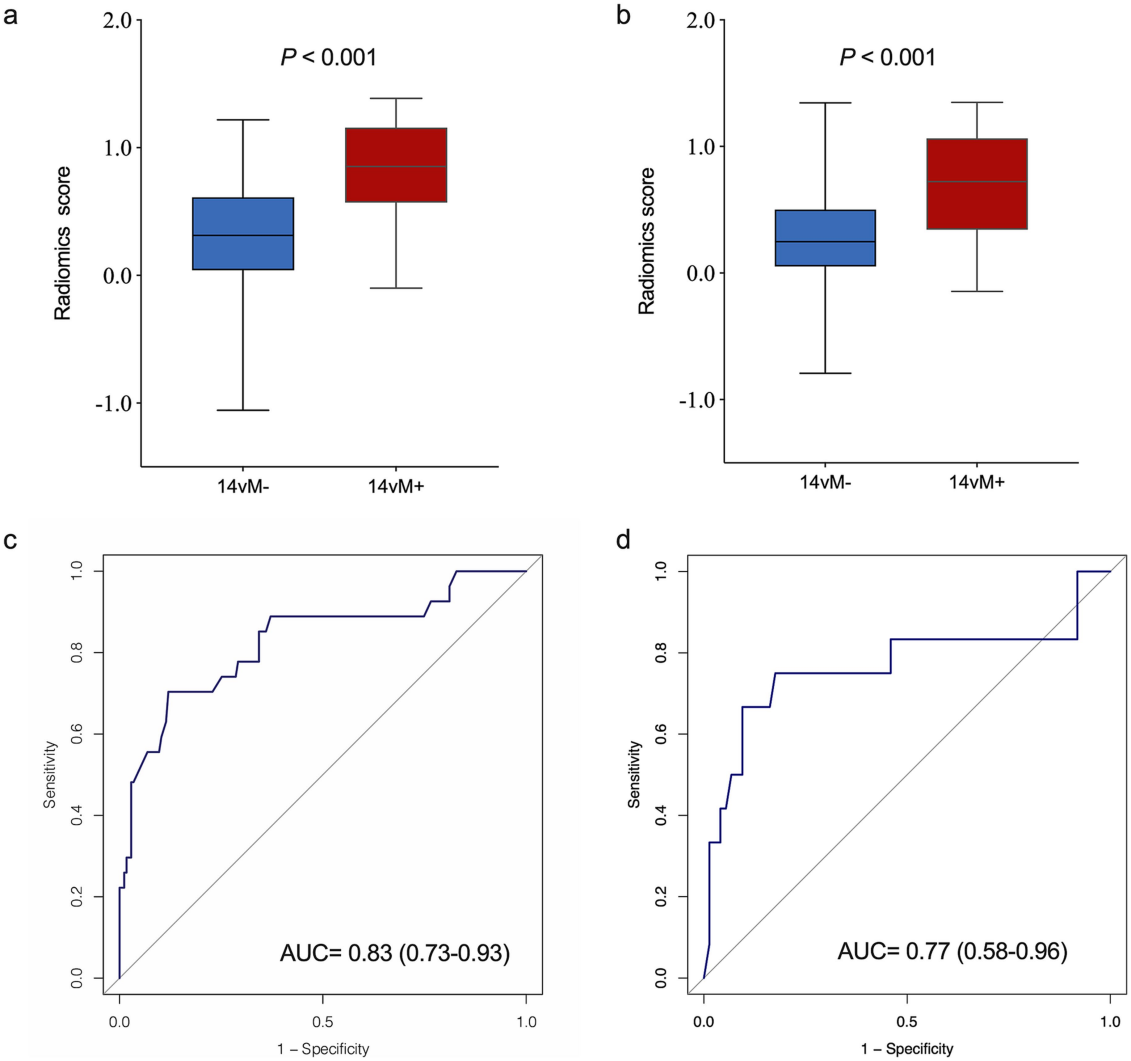
Comparison of radiomics score between No. 14v station lymph node metastasis 14vM− and 14vM+ groups in the training (a) and testing (b) sets. The ROC curves of the radiomics signature in the training (c) and testing (d) sets.

The patients with14vM+ had a significant higher level of *R*-score than the 14vM− patients in both sets (*p* < 0.001, Figures 4a,b). The *R*-score showed favorable performance in both sets with AUCs of 0.83 for the training [95% confidence interval (CI): 0.73–0.93, Figure 4c] and 0.77 for the testing sets (95% CI: 0.58–0.96, Figure 4d).

### Model construction and evaluation

**Figure 5:**
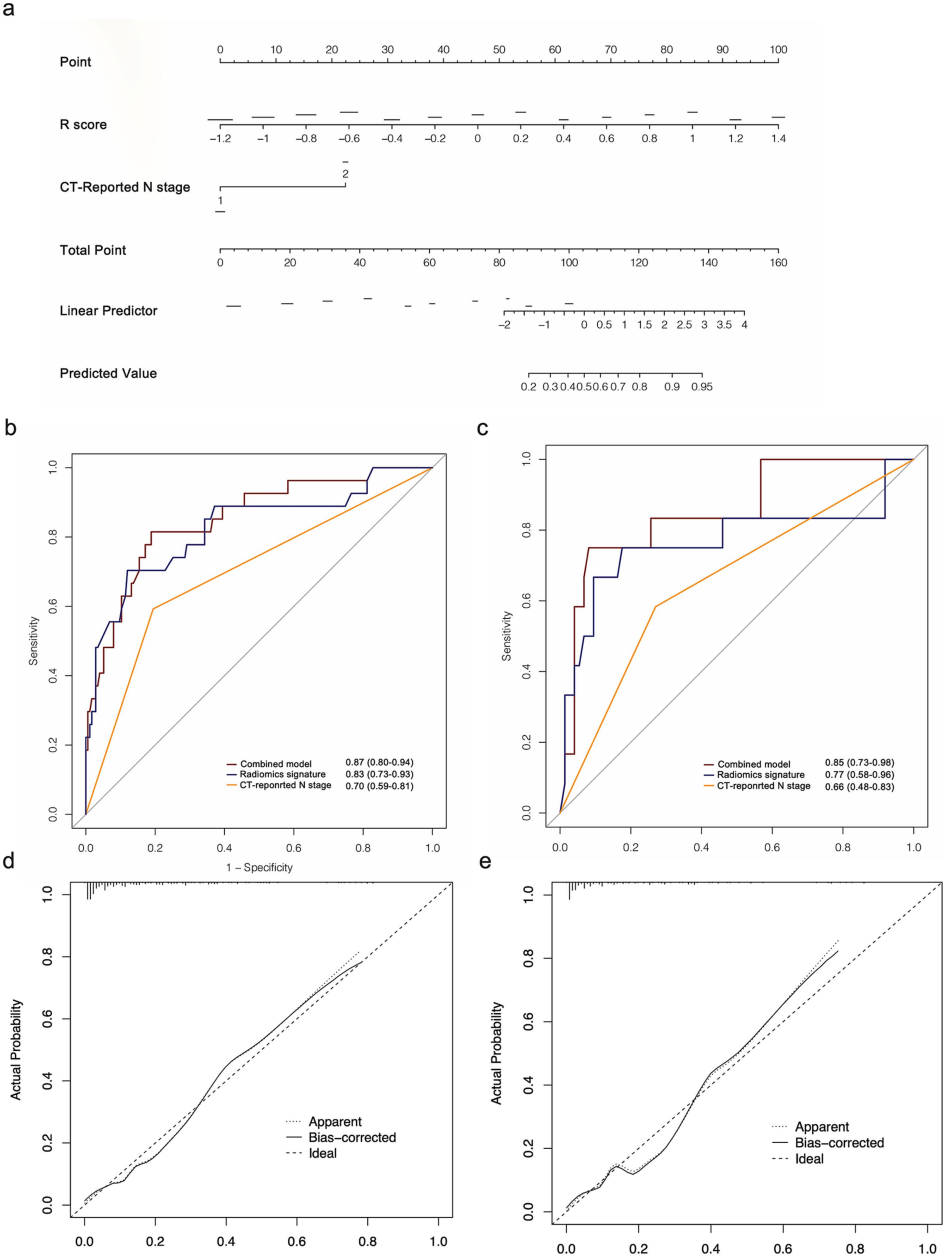
(a) Radiomics nomogram based on radiomics signature and CT-reported N stage. ROC curves of the radiomics nomogram for the prediction of 14vM in the training (b) and testing (c) sets. Calibration curves of the nomogram in the training (d) and testing (e) sets.

The multivariate analysis revealed that radiomics signature, CT-reported and pathological N stage were independent 14vM predictors (Table 3). Because pathological N stage can only be determined postoperatively, we therefore established a combined model by incorporating the *R*-score and CT-reported N stage (Figure 5a).

**Table 3:** Risk factors for 14vM in gastric cancer.

Variable	Univariate logistic regression		Multivariate logistic regression	
	OR (95% CI)	*p*-value	OR (95% CI)	*p*-value
Gender (male vs. female)	0.76 (0.33–1.74)	0.51		
AGE (<65 vs. ≥65)	0.79 (0.28–2.23)	0.66		
Tumor site	0.71 (0.42–1.20)	0.21		
Differentiation	5.82 (0.76–44.47)	0.09		
Pathological T stage	4.72 (1.08–20.71)	0.04	2.62 (0.51–13.52)	0.25
Pathological N stage	11.18 (3.24–38.52)	<0.01	6.24 (1.67–22.97)	<0.01
CT reported N stage	6.03 (2.57–14.17)	<0.01	3.19 (1.17–9.98)	<0.01
CEA	1.35 (0.42–4.30)	0.61		
CA 242	1.43 (0.49–4.14)	0.51		
CA 19-9	1.75 (0.71–4.33)	0.23		
CA 72-4	1.82 (0.81–4.33)	0.14		
Radiomics signature	12.52 (6.53–21.56)	<0.01	8.36 (4.31–18.26)	<0.01

14vM, No. 14v station lymph node metastasis; CEA, carcinoembryonic antigen; CA, carbohydrate antigen.

The combined model had improved AUCs of 0.87 in the training (Figure 5b) and 0.85 in the testing set (Figure 5c). As shown in Table 4, in both sets, the model achieved superior performance compare to the radiomics signature and CT-reported N stage. The calibration curve analysis reflected good fitness of the model (training, *p* = 0.76, Figure 5d; testing, *p* = 0.65, Figure 5e). As shown in Supplementary Figure S2, the DCA also indicated that the model added more benefit than single radiomics and clinical features.

**Table 4:** Predictive performances of radiomics model, radiomics signature and CT-reported N stage.

Model	Training set				Testing set			
	AUC	Accuracy	Sensitivity	Specificity	AUC	Accuracy	Sensitivity	Specificity
Combined model	0.87	0.87	0.78	0.89	0.85	0.84	0.75	0.85
Radiomics signature	0.83	0.86	0.70	0.88	0.77	0.81	0.72	0.84
CT-reported N stage	0.70	0.77	0.59	0.81	0.66	0.77	0.58	0.80

AUC, area under the receiver operating characteristic.

## Discussion

Several studies have reported that 14vM was related to the worse prognosis of GC (5, 7, 8, 26). However, there is still a lack of an effective tool for the prediction of 14vM preoperatively. As far as we are aware, this is the first study to exploit the potential of radiomics in predicting 14vM in GC. In the current study, a ML-based radiomics signature was developed for detecting 14vM in GC patients, showing good predictive power in both sets with AUCs of 0.83 and 0.77, respectively. Moreover, a combined model was developed by integrating the *R*-score with CT-reported N status. The model showed better discrimination power in predicting 14vM.

GC is a highly heterogeneous malignancy disease, and the intratumoral heterogeneity contributes to the risk of metastasis (27). The radiomics methods have been widely used in the characterization of heterogeneity of the tumor microenvironment (28). Instead of focusing on traditional features, radiomics techniques offer a comprehensive understanding of the tumor’s environment and heterogeneity (29). In our study, rather than extracting features from 2D maximum dimension (23), the radiomics features were extracted through 3D VOIs, presenting the whole landscape of tumor bulk. This extraction procedure allowed our model to provide more information for the evaluation of tumor heterogeneity. In addition, the features selected for our model were found to be valuable for characterizing tumor heterogeneity and providing clinicopathological information. Gray level nonuniformity was considered a crucial factor for determining intratumoral heterogeneity (30). NGTDM-busyness and NGTDM-coarseness, which described the pattern and spatial distribution of the voxel intensity of VOI, could also provide information on tumor heterogeneity (31). Features including uniformity and entropy were suggested to have a correlation to the poor prognosis of several tumors (32, 33). It was noted that the size of the tumor is a significant risk factor for 14vM (7). However, as the exact determination of tumor size can only occur after surgery, this parameter was not included in our model. However, one of the radiomic features in our model, the maximum 3D diameter, can enhance information regarding tumor size.

The lymphatic vessel network of the stomach is intricate and characterized by multidirectional flow. Lymphatic vessels from the upper stomach drain into various vessels through the left and posterior gastric arteries, the left inferior phrenic artery and the splenic artery, without connecting to the retro-pancreatic (No. 13) or mesenteric (No. 14) station; while spreading along the common hepatic and superior mesenteric arteries, the lymphatic vessels from the lower stomach drain into the hepatoduodenal ligament (No. 12) and retro-pancreatic (No. 13) nodes station (34). Therefore, the location of GC is related to the risk of LNM in different stations. Previous study revealed that the frequency of 14vM+ was 15.6% in the lower GC, while the frequency was only 4.6% in the upper or middle GC. In patients with 14vM+, 87.8% of the tumor occurred in lower1/3 of stomach (6). Wu et al. (26) also revealed that the 14vM risk was significantly increased in the lower 1/3 of the stomach than in the other sites. Similar to their results, in the current study, 75.8% of the tumor with 14vM+ were localized in the lower stomach.

Tumor infiltration depth correlates positively with the occurrence of lymphovascular invasion and LNM (35, 36). An et al. (5) reported that the risk of 14vM in a tumor that invaded the serosa or deeper was much higher than that confined in mucosal-muscularis propria layers. Eom et al. (7) also showed that the risk of 14vM increased with a higher pathological T stage. In line with prior studies, the depth of tumor invasion was also found to be a significant factor for 14vM. In both sets, the rate of 14vM in T_3–4_ stage was higher than that in T_1–2_ stage patients. These results indicated that the risk of 14vM was increased significantly once tumor invades the serosa. However, the pathological T stage was not determined as a 14vM independent risk factor by multivariable analysis. Thus, this factor was not included in our model.

There are three main lymphatic drainage pathways along the lower stomach: the pathway from lesser curvature (No. 3) or suprapyloric (No. 5) to common hepatic artery (anterosuperior group, No. 8a) lymph nodes, from infrapyloric (No. 6) to No. 8a and from No. 6 to 14v. Eventually, the lymphatic fluid drains into paraaortic lymph nodes (No. 16) (37). Previous studies demonstrated that patients with 14vM tended to have multiple positive nodes in the other stations (6, 26). Furthermore, studies have indicated a strong correlation between 14vM and station 6, suggesting that the station 6 lymph node could serve as the sentinel lymph node for 14vM (6, 8, 26). Therefore, metastasis of other regional lymph nodes along the drainage pathways of 14v can also increase the risk of 14vM. In this study, the rate of 14vM in N_2–3_ patients was 23.9%, which exhibited a more significant increase than in N_0–1_ patients (3.4%). The multivariable analysis further demonstrated that the both CT-reported and pathological N category were independent predictors of 14vM. Since pathological N stage can only be determined postoperatively, we only enrolled CT-reported N stage in our model.

Serum tumor biomarkers, including CEA and CA19-9/24-2/72-4, have been frequently utilized as GC diagnosis biomarkers (38–40). Previous study has reported that CA 72-4 had an added value for LNM predictive accuracy in GC (41). Herein, the correlation between the 4 serum biomarkers with 14vM was investigated, revealing that none of the markers was related to 14vM, which indicates that the traditional serum biomarkers had limited value in detecting 14vM.

Several limitations of this study warrant consideration. Firstly, as a retrospective study, since not all patients received No. 14v dissection, the current analysis may include selection bias. Secondly, variations in scanner parameters can lead to inconsistencies in radiomics features across different institutions. Thirdly, the process of segmentation is both computationally intensive and time-consuming. Lastly, the study was conducted at a single center, indicating a need for larger, prospective multicenter studies to verify the clinical utility of this model.

In summary, the current study highlighted the value of combining ML methods with radiomics technology for predicting 14vM. The radiomics signature showed strong predictive performance 14vM. The nomogram provided a convenient predictive biomarker for preoperative detection of 14vM and help the surgeon to decide whether to add 14v dissection to lymphadenectomy, which may contribute to prognosis improvement of patients.

## Data Availability

The raw data supporting the conclusions of this article will be made available by the authors, without undue reservation.
