# Supplementary material for: A machine learning based radiomics approach for predicting No. 14v station lymph node metastasis in gastric cancer
Source: Front Med (Lausanne). 2024 Oct 18;11:1464632. doi: 10.3389/fmed.2024.1464632 (PMC11527654; doi:10.3389/fmed.2024.1464632)
Supplement: Supplementary file 1 [file Data_Sheet_1.docx]

**Supplementary Materials**

**1. Radiomics feature extraction**

Before feature extraction, CT images were resampled into 1.0× 1.0 × 1.0 mm^3^ resolution using linear interpolation. To standardize the intensity range across scanners, Z-score normalization was utilized. Radiomic features were extracted from the original images of tumors and images transformed by wavelet and Laplacian of Gaussian (LoG) filtration. Original images were wavelet filtered with three directions, resulting eight different combinations of feature. LoG filtration images were generated with sigma=1, 2, 3, 4 and 5 mm.

**2. The threshold values of serum biomarkers**

The carbohydrate antigen (CA) 19-9, CA242, CA72-4, and carcinoembryonic antigen (CEA) were 37 U/mL, 20 U/mL, 6.9 U/mL, and 5.0 μg/mL, respectively.


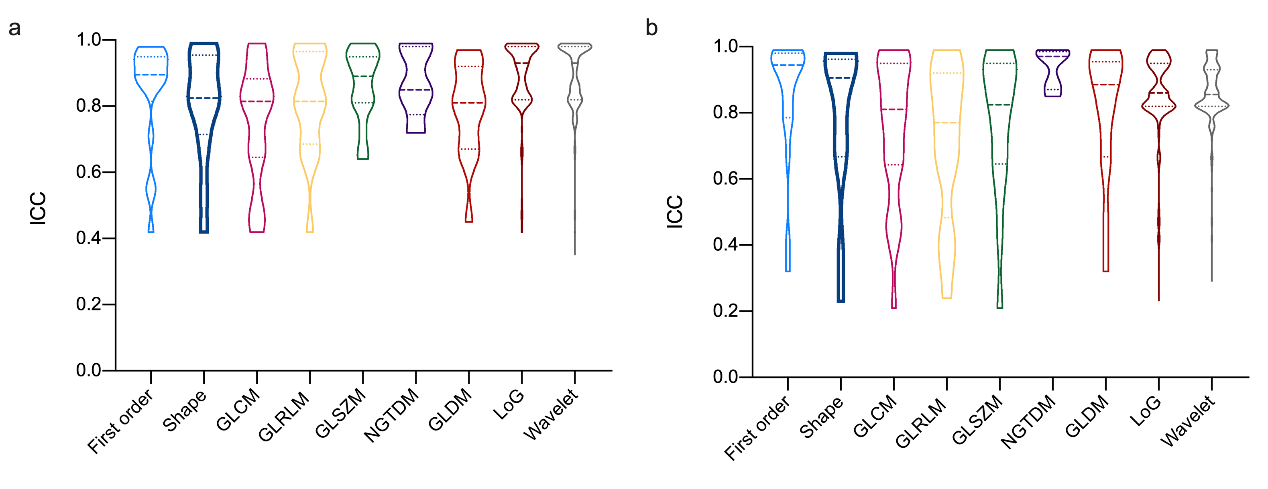


**Fig. S1** Violin plot of ICCs of radiomic features extracted from 9 feature groups. (a) Intraobserver ICCs. (b) Interobserver ICCs. ICCs, intraclass correlation coefficients.

**Fig. S2 ﻿** Decision curve analysis for radiomics signature, CT-reported N stage and combined model. The Y-axis represented the net benefit. The red line represented the combined model. The blue line represented the radiomics signature. The orange line represented the CT-reported N stage.
